# Diagnosis of cystic echinococcosis within a pediatric hospital setting

**DOI:** 10.1128/asmcr.00040-25

**Published:** 2025-05-23

**Authors:** Samuel M. Goodfellow, Andrew Karnaze, Hyunjean Kim, Shengmei Zhou, Leila C. Posch, Cristina Costales

**Affiliations:** 1 Department of Pathology and Laboratory Medicine, Children’s Hospital Los Angeles, Los Angeles, California, USA; 2 Department of Infectious Diseases, Children’s Hospital Los Angeles, Los Angeles, California, USA; 3 Department of Radiology, Harbor-UCLA Medical Center 21640, Torrance, California, USA; 4 Keck School of Medicine, University of Southern California 12223, Los Angeles, California, USA; Vanderbilt University Medical Center, Nashville, Tennessee, USA

**Keywords:** echinococcosis, parasite, infectious pathology, transmission, infectious diagnostics

## Abstract

**Background:**

The diagnosis of early echinococcosis may be difficult due to the small lesion size in the first years of infection and nonspecific symptoms. Here, we discuss a case of cystic echinococcosis at a pediatric center to highlight the challenges in identifying this parasitic disease.

**Case Summary:**

A young female presented to a pediatric hospital with vague abdominal symptoms and an exposure history that aligned with potential echinococcosis. Ultrasound imaging of the liver identified a mixed hyper/hypo-echoic cystic lesion. However, with a negative echinococcal serology result, echinococcus was deemed less likely, and lesion fine-needle aspiration was performed. Cystic echinococcus was confirmed on cytopathology, with features pathognomonic for hydatid cyst. The aspiration procedure did not incur complications; however, the case demonstrates the importance of multifaceted interpretation of infectious testing for echinococcus.

**Conclusion:**

This case highlights the important features of echinococcosis, including the epidemiology, optimal laboratory testing, and management of this complex infection with significant clinical sequela.

## INTRODUCTION

Echinococcosis is a zoonotic parasitic infection caused by cestode (tapeworm) larval stages, which form visceral hydatid cysts primarily in the liver. Most human cases are caused by *Echinococcus granulosus sensu lato* (named due to its broad taxonomic coverage), resulting in cystic echinococcosis (CE) (1). *Echinococcus multilocularis* infection can also occur, resulting in alveolar echinococcosis (AE) (1). Here, we discuss a rare case of CE in our pediatric population while reviewing current practices and guidelines for diagnostics.

## CASE PRESENTATION

**Fig 1:**
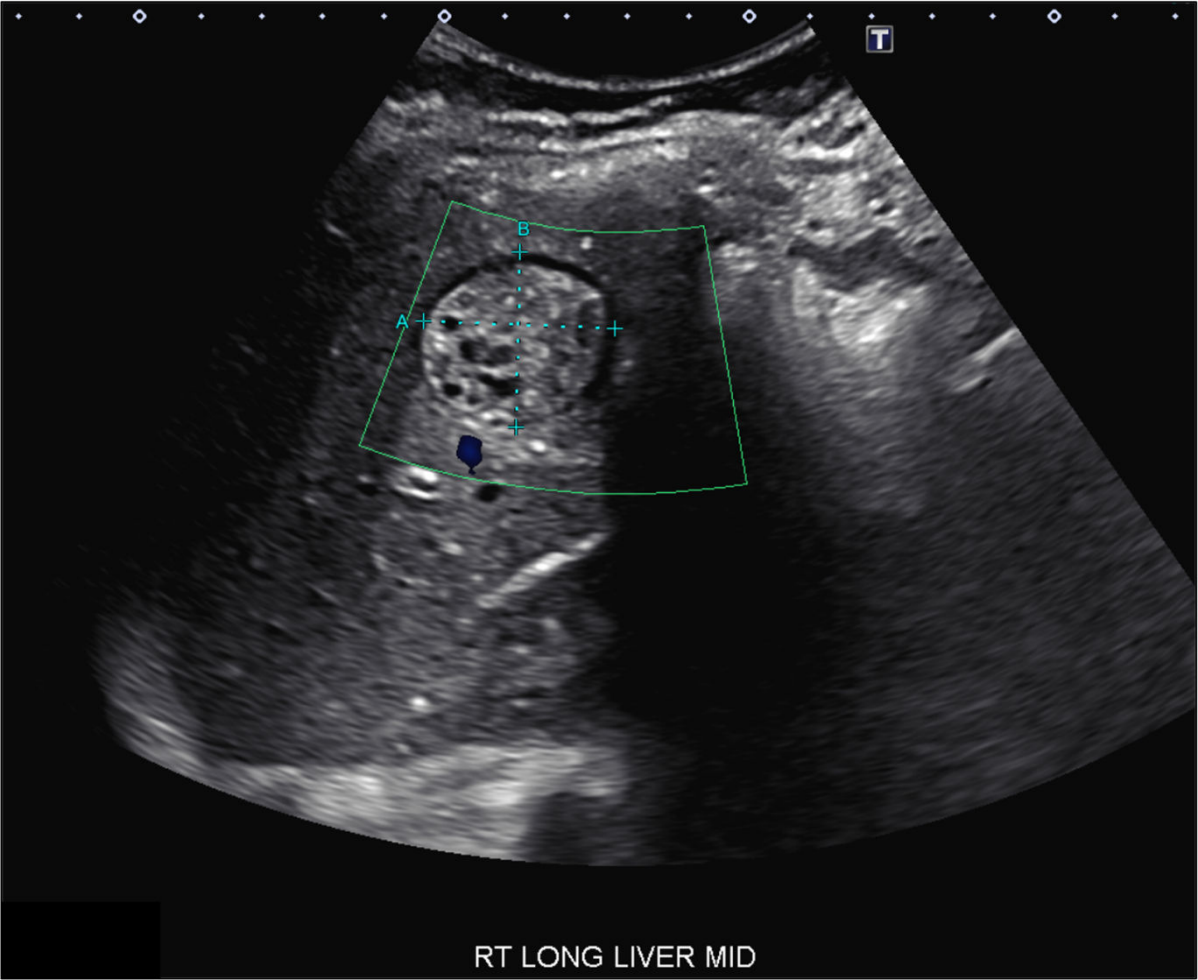
Sagittal color Doppler ultrasound image of the right hepatic lobe demonstrates a 3.1 (A) x 2.9 (B) cm avascular mixed hyper- and hypoechoic lesion with intracystic components.

**Fig 2:**
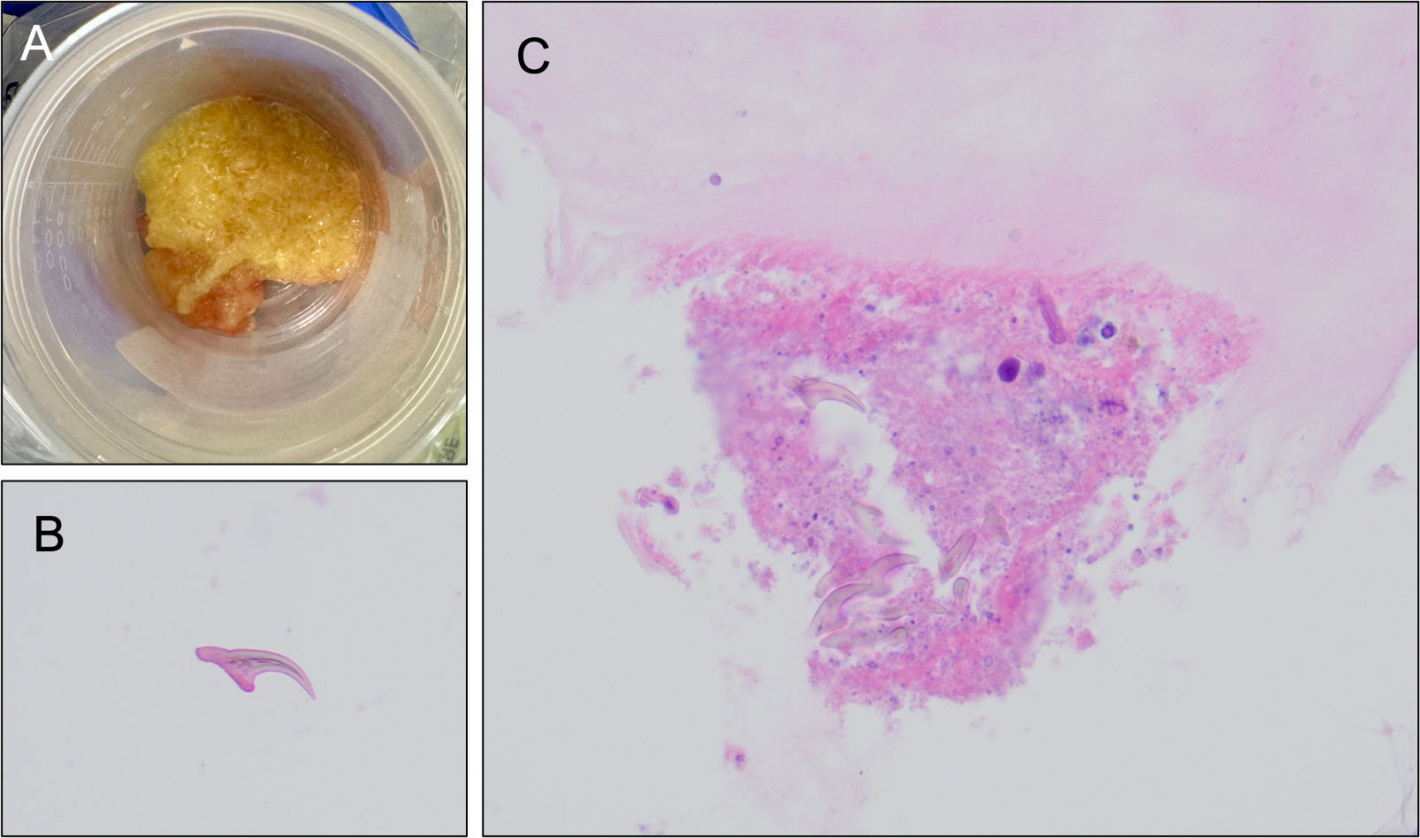
(A) Gross photo of yellow, viscous fluid drained from the liver hydatid cyst. (B) Cell block preparation of aspirated liver cyst fluid showing scattered refractile echinococcal hooklets and (C) degenerating protoscolex of *Echinococcus* with multiple hooklets and acellular laminate membrane of the cyst wall (H&E, 400 x).

A 20-year-old previously healthy female with a history of direct exposure to goats, sheep, and dogs while living on a farm in Guatemala 6 months prior presented with frequent nausea, vomiting, and a reported 20-pound weight loss over 3 weeks. On presentation, she was afebrile, with a heart rate of 94 beats per minute and blood pressure of 125/95 mmHg. Patient was well-appearing, with a benign abdominal examination. Her white blood count was 11.6 K/µL with 73.1% segmented neutrophils (normal range 42.5%–73.2%) and 0.3% eosinophil count (normal range 0.0% to 3.0%). Her C-reactive protein was elevated at 1.9 mg/dL (normal range 0.0–0.9 mg/dL), with slightly elevated AST at 88 (normal range 15–46 units/liter) and ALT at 49 (normal range ≤40). Total bilirubin was within normal limits at 0.89 mg/dL. An ultrasound of the abdomen revealed a 3.1 cm round lesion in the right lobe of the liver that appeared complex (Fig. 1). Further imaging by computed tomography (CT) and magnetic resonance imaging (MRI) confirmed a single, well-defined hepatic cystic lesion measuring 2.9 × 3.1 x 3.5 cm, without significant findings in the lungs. Serologic testing was negative for *Echinococcus granulosus* IgG, Brucella IgG/IgM, Bartonella IgG/IgM, and *Entamoeba histolytica* EIA, raising concern for a potential malignancy. Fine-needle aspiration was performed by interventional radiology for both medical management and diagnostic purposes, with approximately 8 mL of yellow, viscous fluid aspirated (Fig. 2A). Cytospin of the fluid revealed rare refractile hooklets without intact protoscoleces observed (Fig. 2B and C), consistent with echinococcal cyst. A reference laboratory confirmed the diagnosis of *Echinococcus* species on Ova and Parasite exam of the aspirated fluid. She was treated with oral albendazole 400 mg twice daily and discharged home with plans to follow-up with repeat liver function testing, abdominal imaging, and to be seen in the infectious disease clinic to determine the final duration of treatment.

## DISCUSSION

One of the most important factors for echinococcus diagnosis is understanding patient exposure risk and thus the regional epidemiology. CE is found worldwide in rural areas; however, some regions have an increased incidence, such as the Peruvian Andes, southern Chile, the eastern Tibetan plateau, and portions of the Mediterranean region. AE is endemic in North America (northwest Alaska and Canada), Asia, and parts of Europe. Both AE and CE have high endemicity in Central Asia (2). In endemic areas, annual CE incidence ranges between <1 and 200 per 100,000 with a mortality rate of 2% to 4%, while AE is much rarer in humans, with an incidence of 0.03 to 1.2 per 100,000, with a higher mortality rate up to 90% in untreated or insufficiently treated patients (2). Within the United States, there are less than five cases per year with rare reports from Alaska and the Southwest, but most transmissions occurred due to travel in endemic areas (3).

Contact with definitive and intermediate host reservoirs is important for human cases of CE and AE. The primary hosts of *E. granulosus* are canids, with a prevalence of up to 31.9% in these hosts, while livestock ungulates are typical intermediate hosts (sheep, cattle, goats, and camels) with a prevalence of 0.003% to 68.73% depending on the region and population (4, 5). Red foxes and other canids are the primary hosts of *E. multilocularis*, and small mammals (e.g., rodents) are the intermediate hosts. Extensive prevention measures have been implemented globally, including increased sanitary regulations of animal slaughtering. Monthly administration of praziquantel for deworming has also been successful for reducing echinococcosis in canids, but infrequent dosing and the potential for re-infection remain an issue in endemic regions such as China (6).

In CE, human infection occurs when *E. granulosus* cestode eggs are ingested from canid feces and hatch in the human small intestine, releasing hooked oncospheres. These oncospheres penetrate the intestinal wall and enter the portal circulation where they can disseminate throughout the body. Once in organs (most commonly liver or lung, rarely other viscera), oncospheres develop into thick-walled cysts and produce protoscolices (larval forms) and daughter cysts. Visceral cysts thus enlarge over time and can contain multiple liters of fluid with thousands of protoscolices (2). Patients typically have asymptomatic infection for many years until cysts enlarge, and hydatid cysts can reach over 10 cm in diameter in the liver before causing symptoms. The average age at diagnosis varies, but CE is more often diagnosed in adults aged 30–40 years, rather than in children or older populations (7, 8). Symptoms are often vague and depend on the affected organ(s), and thus hepatic and pulmonary symptoms are most common. Patients most commonly report abdominal discomfort, poor appetite, nausea, and vomiting, as with our patient. They may have hepatomegaly or a palpable abdominal mass. Abnormal liver function tests have been shown in only about 40% of infected patients (9). If a cyst ruptures spontaneously or during a procedure, patients can develop systemic reactions, including fever, eosinophilia, urticaria, and potentially lethal anaphylactic reaction due to antigen leakage and development of specific IgE antibodies (2). Cysts can also disseminate after rupture to other sites and organs.

The diagnosis of echinococcosis relies on imaging findings of characteristic cystic lesions in a patient with a history of exposure to typical definitive or intermediate hosts in an endemic area. Ultrasonography, radiography, computed tomography (CT), magnetic resonance imaging (MRI), or fluorodeoxyglucose-positron emission tomography (FDG-PET) have all been used. Specific imaging findings can help differentiate between suspected CE versus AE. CE often has a singular fluid-filled hepatic cyst, while AE causes multiloculated lesions with numerous microcysts. Imaging modalities such as contrast-enhanced ultrasound (CEUS) may improve early diagnostic accuracy of hepatic lesions over conventional ultrasound (10). The World Health Organization Informal Working Group on Echinococcosis (WHO-IWGE) has categorized hepatic cysts for CE as CE1 to CE5, with CE1 and CE2 corresponding to “active stages,” CE3a and b as “transitional stages,” and CE4 and CE5 as “degenerating stages” (2, 11, 12). Smaller cysts or lesions (<2 cm in diameter) present a diagnostic challenge; however, additional imaging methods such as contrast-enhanced ultrasonography can overcome this. These advanced imaging techniques are often inaccessible to the rural populations primarily affected by the disease.

The Centers for Disease Control and Prevention (CDC) and WHO-IWGE recommend imaging as the primary diagnostic tool for CE and AE with the use of serological tests to support the diagnosis (13). Reported serology assay sensitivity and specificity vary between 60% and 90% in CE. Intact, unruptured cysts may not elicit sufficient humoral response for serologic detection, limiting serologic clinical utility in early infection (14). In our patient case, we were unable to detect the infection via serology, potentially leading to a misdiagnosis until aspiration and cytopathology revealed otherwise. Combinational epidemiology risk factors and clinical criteria should be prioritized over serological guidance depending on the stage of infection. Polymerase chain reaction (PCR) testing of the cyst fluid is both highly sensitive and moderately specific for confirming *Echinococcus* species; however, PCR is not commercially available and requires invasive sampling from the cyst itself (2). Limited studies piloting plasma cell-free DNA detection of *Echinococcus* species have also shown low sensitivity. However, this method shows promise as a non-invasive testing alternative; thus, the development of higher-quality and more comprehensive sequence databases remains pending (15).

Treatment of CE depends on classification, size and location of cysts, current symptoms, and associated complications. Options include surgical removal, nonsurgical interventions, chemotherapy, a “watch and wait” approach (usually reserved for degenerating stage cysts), or a combination of these treatments. Chemotherapy with oral benzimidazoles (albendazole and mebendazole) is effective in many patients, with one-third experiencing full cure and up to 30%–50% experiencing regression in size and alleviated symptoms. Antiparasitic treatment is typically continued for at least 1–6 months (14). An alternative option for treatment is the percutaneous aspiration, injection of protoscolecides, and reaspiration (PAIR) technique. Intraoperative manipulation of the cysts can lead to spillage of protoscolex-rich fluid, which increases the risk of anaphylaxis and of recurrence. Our patient underwent aspiration to rule out malignancy; however, as per WHO recommendations, aspiration can also be used for clinical management of CE lesions. Lastly, the “watch and wait” approach can be used if symptoms are manageable to avoid invasive procedures and if medication is not desired. There are currently no studies comparing treatments, and thus there no standardized guidelines that are universally accepted. For CE, long-term follow-up is recommended due to the risk of recurrence after surgery and treatment (2).

Based on our patient’s prior geographic location (Central America), exposure history to livestock, single cystic lesion in the liver, and detection of degenerated protoscoleces from the cyst fluid aspirate, a diagnosis of CE caused by *Echinococcus granulosus sensu lato* was made. With patient endemic exposure and lesion characteristics, echinococcosis was high on the initial differential prompting serologic testing. Although antibody testing for *Echinococcus granulosus* was negative in this case, it is important to note that serology has low sensitivity in patients with relatively small, unruptured cysts and therefore cannot reliably exclude the diagnosis. Based on initial results from the aspirated fluid, the patient was started immediately on antihelminth therapy and has done well. She did not experience any complications from her procedure; however, caution is essential when performing any invasive procedure in suspected cases of echinococcal cyst to avoid potentially life-threatening complications from spillage of cyst contents.

### Key points

- Echinococcosis is a zoonotic parasitic infection that can incubate for many years after exposure before triggering clinical symptoms.
- Imaging methods and serology can confirm the diagnosis, but serology shows insufficient sensitivity to rule out the disease.
- Cytopathology findings of echinococcal (hydatid) cysts are pathognomonic, when available.
